# A Supramolecular Stabilizer of the 14‐3‐3ζ/ERα Protein‐Protein Interaction with a Synergistic Mode of Action

**DOI:** 10.1002/anie.201914517

**Published:** 2020-02-11

**Authors:** Alba Gigante, Eline Sijbesma, Pedro A. Sánchez‐Murcia, Xiaoyu Hu, David Bier, Sandra Bäcker, Shirley Knauer, Federico Gago, Christian Ottmann, Carsten Schmuck

**Affiliations:** ^1^ Department of Organic Chemistry University of Duisburg Essen Universitätstr. 7 45141 Essen Germany; ^2^ Department of Biomedical Engineering Eindhoven University of Technology P.O. Box 513 5600 MB Eindhoven The Netherlands; ^3^ Departamento de Ciencias Biomédicas Universidad de Alcalá 28805 Alcalá de Henares Spain; ^4^ Centre for Medical Biotechnology University of Duisburg Essen Universitätstr. 7 45141 Essen Germany; ^5^ Present address: Institute of Theoretical Chemistry Faculty of Chemistry University of Vienna Währinger Str. 17 1090 Vienna Austria

**Keywords:** 14-3-3, ERα, protein-protein interaction, stabilizers, supramolecular systems

## Abstract

We report on a stabilizer of the interaction between 14‐3‐3ζ and the Estrogen Receptor alpha (ERα). ERα is a driver in the majority of breast cancers and 14‐3‐3 proteins are negative regulators of this nuclear receptor, making the stabilization of this protein‐protein interaction (PPI) an interesting strategy. The stabilizer (**1**) consists of three symmetric peptidic arms containing an arginine mimetic, previously described as the GCP motif. **1** stabilizes the 14‐3‐3ζ/ERα interaction synergistically with the natural product Fusicoccin‐A and was thus hypothesized to bind to a different site. This is supported by computational analysis of **1** binding to the binary complex of 14‐3‐3 and an ERα‐derived phosphopeptide. Furthermore, **1** shows selectivity towards 14‐3‐3ζ/ERα interaction over other 14‐3‐3 client‐derived phosphomotifs. These data provide a solid support of a new binding mode for a supramolecular 14‐3‐3ζ/ERα PPI stabilizer.

## Graphical abstract

**A stabilizer** of the interaction between the Estrogen Receptor alpha (ERα), a driver in the majority of breast cancers, and 14‐3‐3ζ, a negative regulator of ERα, is developed as anti‐cancer strategy. The stabilizer of this protein‐protein interaction (PPI) consists of three symmetric peptidic arms and stabilizes the 14‐3‐3ζ/ERα interaction synergistically with the natural product Fusicoccin‐A.

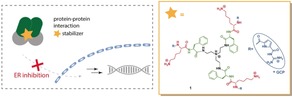

Members of the 14‐3‐3 protein family are regulatory adapter elements in intracellular signaling pathways by means of recognition of proteins that contain phosphorylated Ser/Thr residues like those implicated in the MAPK (mitogen‐activated protein kinase) pathway.^1^, ^2^ As a measure of their relevance, more than 300 potential binding partners of this family have been identified. Many of these have been shown to be involved in disease.^3^ This fact supports the notion that several 14‐3‐3 protein‐protein interactions (PPIs) can be attractive therapeutic targets.^4^, ^5^

The recent elucidation of crystal structures of different ligands in complex with 14‐3‐3 proteins opens up opportunities to develop new supramolecular ligands to modulate 14‐3‐3 PPIs.^6^, ^7^, ^8^, ^9^, ^10^, ^11^, ^12^, ^13^, ^14^ Crystal structures of different 14‐3‐3 isoforms bound by phosphorylated peptides derived from effectors such as C‐Raf,^15^, ^16^ ExoS^19^, ^20^ and PKC‐ϵ^21^ improved our understanding of the structural principles that govern these PPIs. In general, 14‐3‐3 binds phosphomotifs through its Arg‐Arg‐Tyr triad in a central binding groove, which can be broadly categorized as internal mode‐I/II, RSX{**pS**/**pT**}XP, or C‐terminal mode‐III, {**pS**/**pT**}X‐*COOH* motifs.^23^

This study focuses on the interaction of 14‐3‐3*ζ* with the mode‐III motif of the Estrogen Receptor α (ERα), where in 2013, de Boer and co‐workers identified the natural product Fusicoccin‐A (FC‐A) as the first 14‐3‐3ζ/ERα stabilizer.^24^ Stabilization of this interaction was found to reduce ERα dimerization and ERα/DNA interaction, resulting in down‐regulation of ERα‐controlled gene expression and decreased cell growth, validating the approach of PPI stabilization for 14‐3‐3/ERα by small molecules as an alternative inhibitory strategy for ERα activity (Figure 1). Consequently, the identification of synthetically more accessible and more selective stabilizers, but with the same stabilizing potential as FC‐A, is highly desirable.

**Figure 1:**
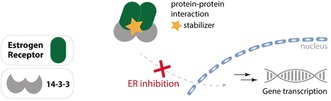
Simplified representation of the cellular pathway and the rationale for small‐molecule stabilization of the interaction between 14‐3‐3 and the Estrogen Receptor (ER) therein.

Recently, we have published several supramolecular ligands as stabilizers of the 14‐3‐3ζ/C‐Raf and 14‐3‐3ζ/Tau interactions. All of these molecules contain the guanidinocarbonylpyrrole (GCP) moiety; an arginine mimetic described by our group.^25^, ^26^, ^27^, ^28^ This non‐proteinogenic amino acid generates very stable ion pairs with oxoanions such as carboxylates and phosphates. In addition, the previously described stabilizers presented a multivalent effect, so that those containing 3 or 4 arms stabilized the best. Based on these characteristics we selected compound **1** (Figure 2 a) from our in‐house library to be evaluated as a stabilizer of 14‐3‐3 PPI. Although **1** contains 3 arms with one GCP motif each, it is smaller and its scaffold, as well as its peptide sequence, are different from the previously reported stabilizers. We analysed the stabilization activity of **1** towards 14‐3‐3ζ/effector interactions by fluorescence anisotropy, at a protein concentration for which the complex is initially not formed (Figure S1 a, Supporting Information). Selected binding partners that were included are: Tau‐, C‐Raf‐ and Cdc25B‐derived motifs (mode‐I/II) and the C‐terminal sequences of ERα and TASK3 (mode‐III). The titration of **1** suggested preferential stabilization of the 14‐3‐3ζ/ERα interaction over the other clients (Figure S1 b).

**Figure 2:**
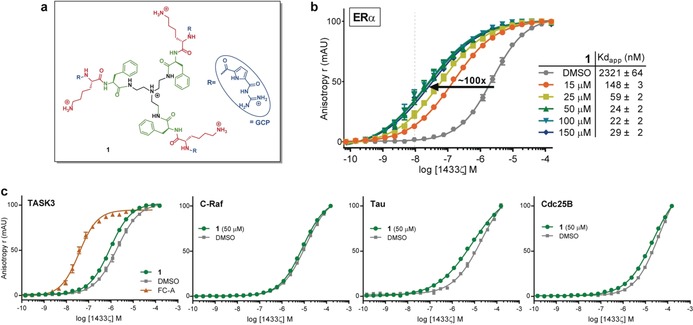
Supramolecular stabilizer of the 14‐3‐3ζ/ERα interaction. (a) Chemical structure of compound **1**. (b) Apparent *K*_D_ of 14‐3‐3ζ/ERα interaction as observed from 14‐3‐3ζ titrations to fluorescein‐labeled ERα‐derived phosphopeptide in the presence of increasing concentrations of **1**, resulting in a stabilization of the PPI of up to 100‐fold. (c) Apparent *K*_D_ of 14–3‐3ζ and representative phospho‐motifs of other clients (TASK3; mode III, and C‐Raf, Tau and Cdc25B; mode I/II) in the absence and presence of 50 μm of **1**.

We determined the stabilization activity of **1** towards the 14‐3‐3/ERα complex by titrating the ERα‐derived phosphopeptide with 14‐3‐3ζ in the presence of increasing concentrations of **1**, resulting in an increased affinity of the PPI up to 100‐fold (Figure 2 b). We observed this highest stabilization for all concentrations of **1** from 50 μm and up (*K*_D_,_app_≈20 nm), where we ran into the detection limit of this assay (since 20 nm ERα peptide was used). A concentration of 50 μm of **1** was subsequently used in titrations of 14‐3‐3ζ to TASK3, C‐Raf, Tau and Cdc25B (Figure 2 c), for which no significant stabilization effect was found. A 50‐fold stabilization by FC‐A was observed for the 14‐3‐3ζ/TASK3 interaction.

We next set out to analyse the mode of action for stabilization of 14‐3‐3ζ/ERα by compound **1** in more detail. Interestingly, we observed a synergistic effect for stabilization by **1** and FC‐A (Figure 3). ERα phosphopeptide was titrated with 14‐3‐3ζ in the presence of **1**, FC‐A, or both. The presence of 20 μm of compounds individually resulted in an enhanced affinity of the protein complex of 20‐ or 37‐fold, respectively, whereas a combination revealed a significantly increased stabilization of ≈200‐fold (Figure 3). This suggested that **1** and FC‐A occupy different sites on the protein‐protein complex, allowing a synergistic mode of action.

**Figure 3:**
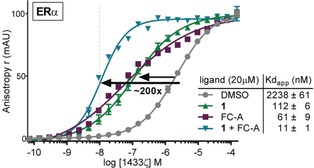
Synergistic stabilization by compound **1** and Fusicoccin‐A (FC‐A) of the 14–3‐3ζ/ERα interaction. At 20 μm compound, a 20‐ and 37‐fold increase of PPI affinity is observed for **1** and FC‐A individually, respectively, whereas when combined resulting in a ≈200‐fold increase.

To obtain structural insights explaining the molecular basis for selectivity of **1** towards the 14‐3‐3ζ/ERα complex and synergistic activity of **1** and FC‐A, we undertook several unsuccessful attempts to crystallize the 14‐3‐3ζ/ERα/**1**/FC‐A complex. For this reason, we built a molecular model by combining automated docking and several independent restrained molecular dynamics (MD) simulations (detailed description provided in the Supporting Information) using as coordinates for the complex one of our previously solved crystal structures (PDB entry 4JDD). In our model, two of the three arms of **1** interact with each of the two ERα peptides located in each 14‐3‐3ζ monomer (Figure 4). The GCP groups were found to interact with two of the phosphate oxygens of the phosphorylated threonine residue of ERα (pThr594), whose electron lone pairs are compromised with the surrounding side chains. Indeed, the GCP moiety has been proved previously to be an excellent phosphate binder. Noteworthy, the GCP guanidinium moiety in our model partially overlaps with the Arg370 side chain of TASK3 bound to 14‐3‐3ζ (PDB entry 3SML). This might explain why **1** does not stabilize the interaction of the latter in the presence of TASK3, whereas FC‐A does not differentiate between mode‐III motifs. On the other hand, the Lys side chain of **1** binds to the C‐terminal residues of ERα, a typical feature of mode‐III binding effectors. This interaction is absent if there are additional residues at the C‐terminus (e.g., binding mode‐I/II effectors such as Tau, C‐Raf or Cdc25B).

**Figure 4:**
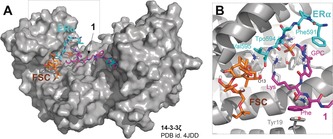
Molecular model for the complex 14–3‐3ζ/ERα/FC/**1**. A) Compound **1** extends its arms to both effector binding sites. B) Detail of one effector binding site in 14‐3‐3ζ.

Consequently, whereas the GCP of **1** is likely responsible for the recognition of the phosphate group and does not allow an arginine side chain in close proximity, the interaction of the side chain of the lysine of **1** is responsible for the selectivity towards mode‐III effectors. The third residue, Phe, is found in proximity to the central binding groove of 14‐3‐3, like the third arm of **1**, which is projected perpendicularly to ERα. In light of our model, the binding modes of **1** and FC‐A do not appear to be mutually exclusive. In order to explain the observed synergistic activity of **1** and FC‐A (Figure 3), we ran additional MD simulations of the 14‐3‐3ζ/ERα complex in the presence of **1** but in the absence of FC‐A. No direct interaction between **1** and FC‐A was found in our simulations (Figure S3). However, ERα mediates the interaction of both organic compounds through its C‐terminus. Interestingly, the absence of FC‐A deeply increased the mobility of the ERα peptide along the MD simulations (RMSd values >3 Å, Figure S4 A). As an example, the residue Ala 593 explored novel regions of the Ramachandran plot in most of the simulations (Figure S4 B). As a consequence of these conformational changes, the binding energy values of ERα to 14‐3‐3ζ dropped down significantly (Figure S4 C). Therefore, the synergistic effect observed for stabilization by **1** and FC‐A towards the ERα/14‐3‐3ζ PPI complex originates from both a direct interaction between **1**, ERα and FC‐A, as well as conformational restriction posed by FC‐A on the peptide residues, resulting in enhanced binding affinity of ERα.

Further support for our molecular model was obtained from a derivative of **1** where its GCP motifs were replaced by Lys (**2**), which resulted in a decrease of activity (80‐ to 12‐fold stabilization, Figure 5), illustrating the importance of the GCP motif. Additionally, replacing the Phe and Lys residues in the peptidic side chains of **1** by Ala (**3** and **4**) also resulted in a drop of stabilization (20‐ and 14‐fold stabilization, Figure 5). According to our model, the presence of Phe residues in **1** would reduce the degree of freedom of the stabilizer by anchoring **1** to the central pore of 14‐3‐3ζ, and thereby, this would stabilize the interactions within the protein. In contrast, Lys residues in **1** provide selectivity. Lastly, we permutated Phe↔Lys in **1** (**5**), which also resulted in a drop of activity (to 30‐fold stabilization), highlighting the optimal geometry of **1** and the importance of its side chain residues.

**Figure 5:**
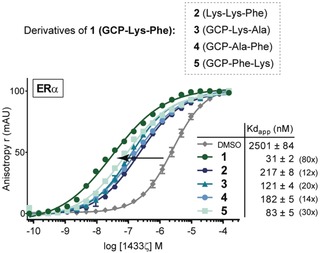
Protein titration data for stabilization of the 14‐3‐3ζ/ERα interaction by derivatives of **1**.

Finally, we measured the cytotoxicity of **1** in Hela and 293T cell cultures to analyze the safety of the compound for future biological assays. Virtually 100 % of cell viability in both cell lines was observed in the presence of 150 μm of **1** after 24 h incubation (Figure S3).

In summary, we have discovered a multivalent synthetic stabilizer of the 14‐3‐3ζ/ERα interaction and proposed the molecular mode of action for its potency and selectivity towards this PPI based on a molecular model, supported by experimental data. Moreover, the binding site of **1** was found to be adjacent to that of FC‐A, which supported the synergistic stabilization effect of these compounds, resulting in an increase of the apparent affinity of the 14‐3‐3ζ/ERα interaction of up to ≈200‐fold. This study provides a valuable new compound as tool for further studies to analyze different events in which the 14‐3‐3ζ/ERα interaction is involved and additionally opens the door for further investigations of synergistic binding modes with the natural product FC‐A for 14‐3‐3 protein‐protein interaction modulation as a strategy for selectivity.

## Conflict of interest

The authors declare no conflict of interest.

## Supporting information

As a service to our authors and readers, this journal provides supporting information supplied by the authors. Such materials are peer reviewed and may be re‐organized for online delivery, but are not copy‐edited or typeset. Technical support issues arising from supporting information (other than missing files) should be addressed to the authors.

Supplementary
