# Supplementary material for: A Supramolecular Stabilizer of the 14‐3‐3ζ/ERα Protein‐Protein Interaction with a Synergistic Mode of Action
Source: Angew Chem Int Ed Engl. 2020 Feb 11;59(13):5284–7. doi: 10.1002/anie.201914517 (PMC7155037; doi:10.1002/anie.201914517)
Supplement: Supplementary file 1 — Supplementary [file ANIE-59-5284-s001.pdf]

Angewandte Chemie
Eine Zeitschrift der Gesellschaft Deutscher Chemiker

Supporting Information

A Supramolecular Stabilizer of the 14-3-3ζ/ERα Protein-Protein Interaction with a Synergistic Mode of Action

Alba Gigante, Eline Sijbesma, Pedro A. Sánchez-Murcia, Xiaoyu Hu, David Bier, Sandra Bäcker, Shirley Knauer, Federico Gago, Christian Ottmann, and Carsten Schmuck*[†]

anie_201914517_sm_miscellaneous_information.pdf

Supporting Information

1. Protein expression and purification
2. Fluorescence Polarization assays
3. Computational studies
4. Toxicity assay
5. Chemistry
    a. General remarks
    b. Synthesis and characterization
    c. ^{1}H- and ^{13}C-NMR spectra of undescribed compounds
    d. HPLC purity
6. References

1. Protein expression and purification

Escherichia coli Rosetta (DE3) cells (Merck, Nottingham, England) transformed with the cloned vector were used to inoculate 50 mL Luria-Bertani (LB) medium (ampicillin 100 μg/mL). The culture was grown at 310 K, 140 rpm. The pre-culture was used to inoculate a 2 L Terrific Broth (TB) culture (ampicillin 100 μg/mL). The culture was shaken at 140 rpm at 310 K until an OD_{600} of 0.4-0.6 was reached. Protein expression was started by adding 0.4 mM IPTG to the culture. The culture was incubated at 298 K for 12 h and then harvested by centrifugation.
The bacterial pellet was re-suspended in buffer (50 mM Tris-HCl, 300 mM NaCl, 5% glycerol, 10 mM imidazole, 0.5 mM TCEP and 1 mM PMSF pH 8.0). The cells were lysed using a microfluidizer. The lysate was cleared using an AKTA Purifier system (GE Healthcare, Freiburg, Germany) and Ni-NTA resin (GE Healthcare, Freiburg, Germany) according to the manufacturer's manual. The resin was washed in buffer (50 mM Tris-HCl, 500 mM NaCl, 5% glycerol, 25 mM imidazole and 0.5 mM TCEP pH 8.0). The protein was eluted (buffer 50 mM HEPES, 200 mM NaCl, 5% glycerol, 250 mM imidazole, 0.5 mM TCEP pH 8.0) and dialyzed against 25 mM HEPES, 100 mM NaCl, 10 mM $MgCl_2$, 1 mM beta-mercaptoethanol pH 7.0. The 14-3-3 protein was concentrated to 50 mg/mL, aliquoted, flash-frozen in liquid nitrogen, and stored at 193 K.

2. Fluorescence Polarization assays

The Fluorescence Polarization measurements were done on a filter-based microplate reader (Tecan Infinite F500). The FAM-labeled peptides were measured with an excitation wavelength of λ_{ex}: 485 ± 20 nm and at an emission wavelength of λ_{em}: 535 ± 25 nm. All measurements were done with an integration time of 50 μs in black, flat-bottom 384 microwell plates (Corning, # 4514/3676). Samples were diluted in buffer (10 mM HEPES, 150 mM NaCl, 0.1% (v/v) Tween 20 and 0.1% (m/v) BSA). GraphPad Prism 5.03 software was used for data analysis. Peptides were supplied by Caslo, Denmark and sequences were as follows:

ERα: (5-FAM)-KYYITGEAEGFPApTV-*COOH*;
TASK3: (5-FAM)-RRKpSV-*COOH*;
C-Raf: (5-FAM)-RQRSTpSTPNVH-$CONH_2$;
Tau: (5,6-FAM)-RTPpSLPTGGGSGGSGGGSKCGpSLGNIHHK-$CONH_2$;
Cdc25B: (5-FAM)-QRLFRSPpSMPCSVIR-$CONH_2$.

An initial titration experiment was performed on client-derived fluorescein-labelled phosphopeptides, titrated with of 14-3-3ζ protein in order to select an appropriate protein concentration for the subsequent stabilization experiments by compound titrations for each client individually (at the start of their binding curves), as previously described.[1,2] All protein titration data is normalized to correct for variations in peptide length. Compound **1** was subsequently titrated on the fluorescently labelled peptides and 14-3-3ζ at the selected concentrations (Figure S1). The anisotropy values were plotted against the logarithmic concentration of the compounds and the resulting curve was fitted to a four-parameter logistic model (4PL) to obtain EC_{50} values. Protein titrations were repeated for fluorescein-labeled ERα peptide (20 nM) in the presence of compound **1, 2, 3, 4** and **5**. These compounds were all additionally titrated (from 1 mM) to ERα peptide (20 nM) in the presence (300 nM) or absence of 14-3-3ζ. These results indicate that these compounds do not bind to the peptides alone. (Figure S2).

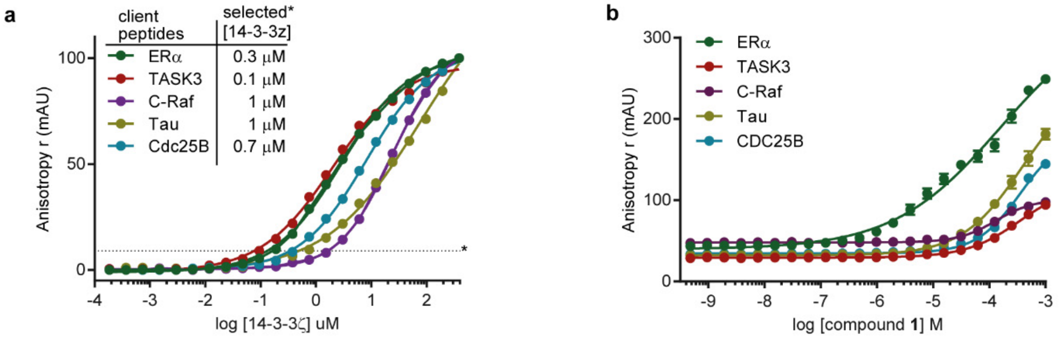

Figure S1. a) Direct binding as observed from titrations of 14-3-3ζ to various client-derived fluorescein-labelled phosphopeptides (ERα, TASK3, C-Raf, Tau and Cdc25B). Selected protein concentrations for compound titration experiments are indicated for each client. b) Titration data for **1** to the phosphopeptides and 14-3-3ζ.

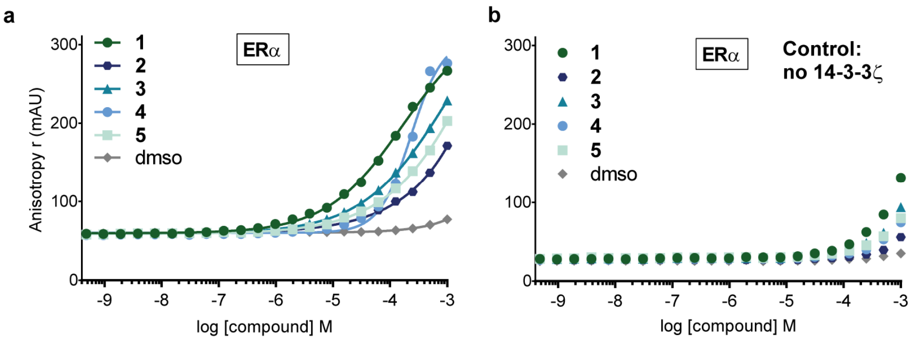

Figure S2. Titration data for ERα and 14-3-3ζ. a) Compound (**1**, **2**, **3**, **4**, **5**) titrations into fluorescein-labelled ERα phosphopeptide in the presence of 300 nM 14-3-3ζ (a), or the control, without protein (b).

3. Computational studies

The molecular models for the complexes **1**:14-3-3ζ were prepared in three general steps. First, probe **1** was docked into the binding sites of the modulator protein using AutoDock4.0[3] in the presence of the 5-mer peptide 591-FPATpV-595 of the human Estrogen receptor Peptide alpha (ERα), which contains the phosphorylated Thr594 (pT), and fusicoccin (FC-A). The Cartesian coordinates of the trimeric ERα/14-3-3ζ/FC-A complex were taken from its crystal structure at a resolution of 2.1 A (PDB entry. 4JDD).[4] Complex **1** was divided by arms and docked in an incremental manner, where each of the arms was added gradually to the system. The grid boxes where centered at pT binding site and at the central pore with a size of 60x60x60 Å. 13 torsional degree of freedom were included for **1** and a genetic algorithm (GA) was used for the docking, including 150 number of individuals per population and 20 runs. The GA was performed several times. After visual inspection, the best solution was selected for refinement using force field MD simulation.

The optimized geometries of FC-A, GCP and Tp (named as TPO) and their force-field parameters were computed following the standard method (HF/6-31G**//HF/3-21G) using the RESP ESP charge Derive Server Development.[5] All the atoms were described as AMBER atom type and the point charges were described as RESP charges. After fitting to the atoms the electrostatic potential was computed using the program *antechamber* of AmberTools18.[6] The leaprcff14SB force field was used in all the MD simulations. The MD simulations were run on GPUs using the pmemd.cuda_SPFP module implemented in Amber18.

Both ERα/14-3-3ζ/FC-A/**1** and ERα/14-3-3ζ/**1** complexes were embedded in a truncated octahedral box of ca. 24000 TIP3P water molecules[7] that extended 12 Å away from any solute atom and 21 Na^+ ions were added to ensure charge neutrality. The system was relaxed by energy minimization in three consecutive steps (3 x 5000 cycles), in which after the first 1000 cycles the minimization method was switched from steepest descendent to conjugate graduate. The resulting system was heated from 100 to 300 K during 200 ps with a time step of 0.1 fs and with the position of all the solute atoms restrained with a harmonic constant of 200 kcal mol^{-1} $Å^{-2}$. The Langevin thermostat (friction coefficients 1.0 ps^{-1}) was employed for the temperature regulation and the simulation was run with fixed volume (NVT ensemble). The harmonic restraints were gradually reduced in six steps from 100 to 5 kcal mol^{-1} $Å^{-2}$. Then, the density of the system was equilibrated for 20 ps using a time step of 0.2 fs by fixing the pressure, using the Langevin thermostat with isotropic pressure scaling (NPT ensemble), and allowing the volume of the box to change. 5 independent MD simulations of 100 ns were submitted for each of the systems (5 x 100 ns per system) at 300 K with a time step of 2 fs. To generate different initial conditions, the velocities of the system were randomly assigned during each of the heating steps. A harmonic restraint of 5 kcal mol^{-1} $Å^{-2}$ was imposed on the alpha carbon atoms of the protein and ERα in order to keep the secondary structure. The cut-off distance for the non-bonded interactions was 10 Å and periodic boundary conditions were used. Electrostatic interactions were treated by using the smooth particle mesh Ewald (PME) method[8] with a grid spacing of 1 Å. The SHAKE algorithm[9] was applied to all bonds involving hydrogen atoms. The trajectories were analyzed with the module *cpptraj* of AmberTools18 and the binding energies ΔG_{bind} of the 5-mer peptide 591-FPATpV-595 of ERα/14-3-3ζ/FC-A/**1** computed with MM-ISMSA.[10]

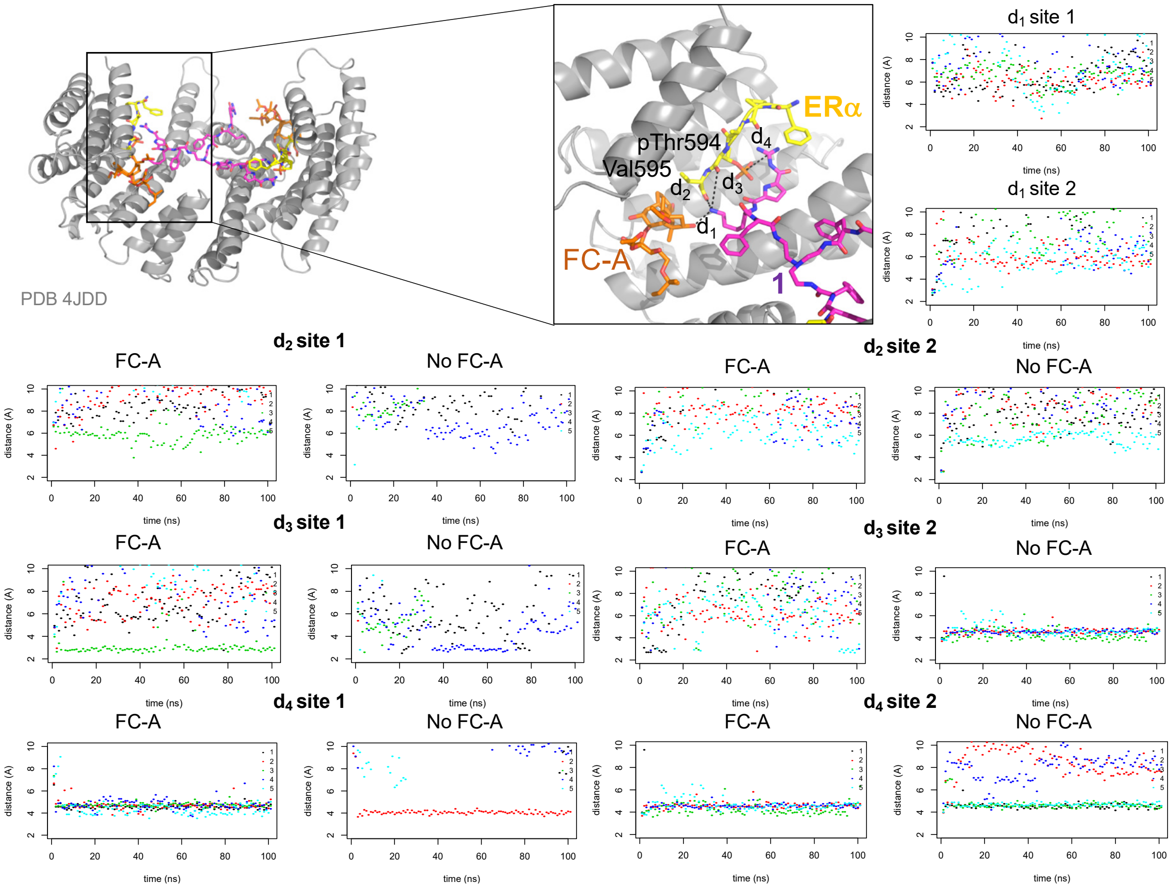

Figure S3. Evolution of distances d_1-d_4 (Å) along 5 independent 100ns-restrained MD simulations of each of the ERα/14-3-3ζ/FC-A/**1** (labeled as FC-A) and ERα/14-3-3ζ/**1** (no FC-A) complexes in solution. In each case, both binding sites in 14-3-3ζ where monitored. The alpha carbon atoms of the protein where restrained with a harmonic force constant of 5 kcal mol^{-1} Å^{-2}.

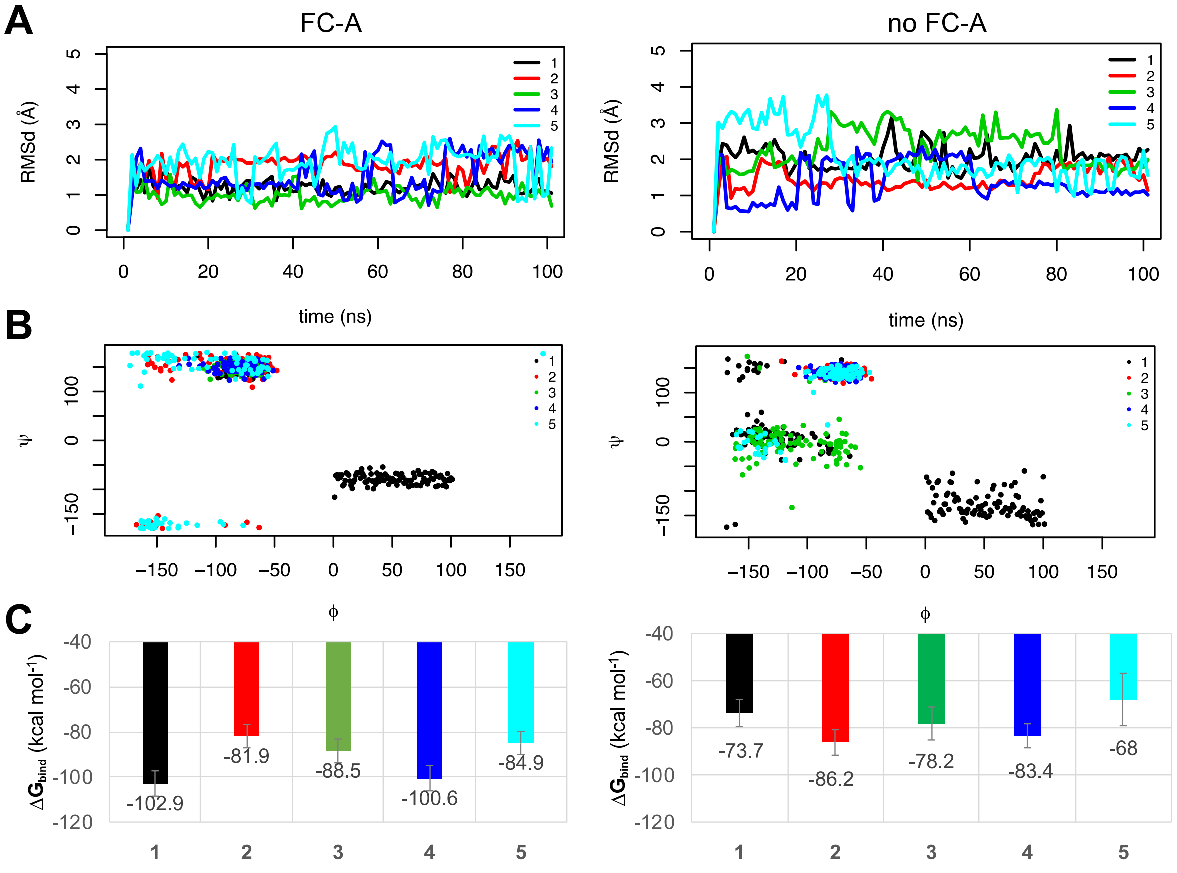

Figure S4. Analysis of the binding of ERα to 14-3-3ζ in the presence of (FC-A) or in the absence (no FC-A) of FC-A. In all cases, the MD simulations were carried out in the presence of **1**. (A) Root-mean-squared deviation (Å) of ERα (only heavy atoms) along the five independent 100ns-MD simulations. (B) Phi and Psi angles (deg) of Ala593 of ERα. (C) Mean value and standard deviation of the binding energies (kcal mol^{-1}) of ERα to 14-3-3ζ.

4. Cytotoxicity assay

The relative *in vitro* cytotoxicities of compound **1** against 293T normal cells and HeLa cancer cells were assessed by using the Alamar Blue assay. Briefly, the cells were seeded in 96-well plates at a density of 1×10^5 cells per well in 100 μL of DMEM containing 10% fetal bovine serum, supplemented with 50 U·mL^{-1} penicillin and 50 U·mL^{-1} streptomycin, and cultured in 5% CO_2 at 37 °C for 24 h. Then, compound **1** with different concentrations was added into each well, and the two types of cells were further incubated for 24 h.

Subsequently, 10 μL of Alamar Blue solution was added into each well and incubated for another 3 h in 5 % CO2 at 37° C. After that, the medium in each well was transferred to another black 96-well plate, and the fluorescence was measured at 590 nm using a multimode reader (GloMax-Multi+Detection System, *Promega*). Untreated cells in medium were used as the blank control. All

experiments were carried out with three replicates. The cytotoxicity was expressed as the percentage of the cell viability relative to the blank control.

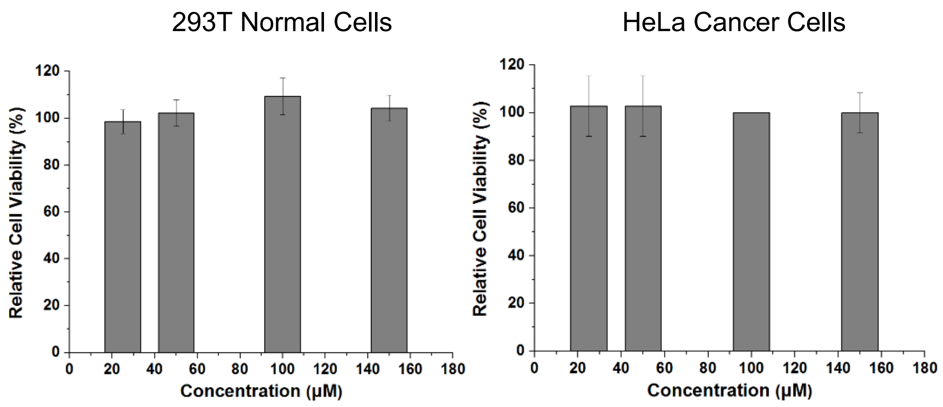

Figure S5. Cytotoxicity of **1** in Hela and 293T cell cultures after 24h incubation.

5. Chemistry

a. General Remarks

Solvents were dried and distilled before use. Millipore water was obtained with a Micropure from TKA. All reactions were carried out in oven dried glassware. Microwave assisted SPPS was carried out with a CEM Discover. Analytical TLC was carried out on SiO_2 aluminum foils ALUGRAM SIL G/UV254 from Macherey Nagel. The analytical "High Performance Liquid Chromatography" (HPLC) was done with Dionex HPLC apparatus: P680 pump, ASI-100 automated sample injector, UVD-340U UV detector, UltiMate 3000 Column Compartment. Commercially available HPLC grade solvents were used as eluents and solvent mixtures are reported in volume percent. Lyophilization was carried out with an Alpha 1-4 2D plus freeze drying apparatus from Christ. Reversed phase column chromatography was done with an Armen Instrument Spot Flash Liquid Chromatography MPLC apparatus with RediSep C-18 Reversed-Phase columns. ^{1}H- and ^{13}C-NMR spectra were recorded on a Brucker DRX 500 MHz spectrometer at ambient temperature. The chemical shifts are reported in parts per million (ppm) relative to the deuterated solvent DMSO-*d6*. The following abbreviations are used for peak multiplicities: s, singlet; d, doublet; m, multiplet; br, broad. High resolution ESI mass spectra were recorded with a Bruker BioTOF III spectrometer. Determination of pH values was carried out with a pH-Meter766 Calimatic from Knick.

b. Synthesis and characterization

Compounds **1**, **2** and **5** were synthesized as described in the literature.[11]

GCP(Boc)-Lys(Boc)-Ala-OH (3a)

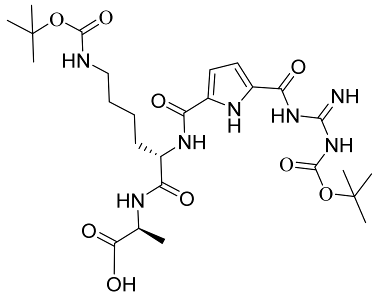

The synthesis of **3a** was carried out accordingly on 2-chlorotriethyl chloride resin (500 mg, 1.55 mmol/g, 0.775 mmol, 1 eq) following the above SPPS procedure. HCTU (641 mg, 1.55 mmol, 2 eq) and DIPEA (540 µL, 3.10 mmol, 4 eq) in DMF (10 mL) were used during the coupling steps. After swallow the resin, Fmoc-Ala-OH (483 mg, 1.55 mmol, 2 eq) with DIPEA (260 µL, 1.55 mmol, 2 eq) in DMF (10 mL) were added. Then, after Fmoc deprotection Fmoc-Lys(Boc)-OH (726 mg, 1.55 mmol, 2 eq) and Boc-GCP-OH (616 mg, 1.55 mmol, 2 eq) were coupled successively. After the cleavage and purification steps, this product was obtained as a white solid (127 mg, 27%, HPLC purity > 90%). ^{1}H-NMR (500 MHz, DMSO-*d*^{6}) δ [ppm]: 1.28 (d, *J* = 7.3Hz, 3H, Ala-CH_3), 1.35-1.43 (m, 13H, 3 x CH_3, Lys-CH_2), 1.46 (m, 9H, 3 x CH_3), 1.57-1.64 (m, 1H, Lys-CH_2), 1.69-1.74 (m, 1H, Lys-CH_2), 2.85-2.91 (m, 2H, Lys-CH_2), 4.16-4.21 (m, 1H, Ala-CH_2), 4.42-4.46 (m, 1H, Lys-CH), 6.75 (m, 1H, Lys-NH), 6.76-6.82 (m, 2H, 2 x GCP-CH_{ar}), 8.29 (d, *J* = 7.2 Hz, 1H, Ala-NH), 8.40 (d, *J* = 8.1 Hz, 1H, Lys-NH), 8.57 (s, 1H, NH), 9.32 (s, 1H, NH), 11.47 (COOH). ^{13}C-NMR (125 MHz, DMSO-*d6*) δ [ppm]: 17.09 (Ala-CH_3), 22.90 (Lys-CH_2), 27.77 (CH_3), 28.26 (CH_3), 29.29 (Lys-CH_2), 31.72 (Lys-CH_2), 40.05 (Lys-CH_2), 47.47 (Ala-CH), 52.37 (Lys-CH), 77.30 ($\underline{C}(CH_3)_3$), 112.87 (GCP-CH_{ar}), 155.52 (Gua-Cq), 158.42 (CO), 159.32 (CO), 171.58 (CO), 173.46 (CO). LRMS (ESI): m/z calculated for $C26H42N7O9^{+}$ $[M+H]^{+}$: 596.3; found: 596.4.

GCP(Boc)-Ala-Phe-OH (4a)

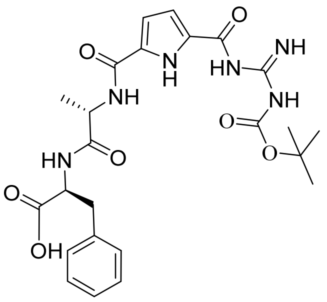

The synthesis of **4a** was carried out accordingly on 2-chlorotriethyl chloride resin (500 mg, 1.55 mmol/g, 0.775 mmol, 1 eq) following the above SPPS procedure. HCTU (641 mg, 1.55 mmol, 2 eq) and DIPEA (540 µL, 3.10 mmol, 4 eq) in DMF (10 mL) were used during the coupling steps. After swallow the resin, Fmoc-Phe-OH (600 mg, 1.55 mmol, 2 eq) with DIPEA (260 µL, 1.55 mmol, 2 eq) in DMF (10 mL) were added. Then, after Fmoc deprotection Fmoc-Ala-OH (483 mg, 1.55 mmol, 2 eq) and Boc-GCP-OH (616 mg, 1.55 mmol, 2 eq) were coupled successively. After the cleavage and purification steps, this product was obtained as a white solid (72 mg, 14%, HPLC purity > 90%). ^{1}H-NMR (500 MHz, DMSO-*d*^{6}) δ [ppm]: 1.26 (d, *J* = 7.1 Hz, 3H, Ala-CH_3), 1.46 (s, 9H, 3 x CH_3), 2.90-2.94 (m, 1H, Phe-CH_2), 3.03-3.06 (m, 1H, Phe-CH_2), 4.40-4.43 (m, 1H, Phe-CH), 4.47-4.52 (m, 1H, Ala-CH), 6.82-6.84 (m, 2H, 2 x GCP-CH_{ar}), 7.14-7.22 (m, 5H, Phe-CH_{ar}), 8.13 (d, *J* = 7.9 Hz, 1H, Phe-NH), 8.47 (d, *J* = 7.6 Hz, 1H, Ala-NH), 8.58 (s, 1H, NH), 9.34 (s, 1H, NH), 11.58 (COOH). ^{13}C-NMR (125 MHz, DMSO-*d6*) δ [ppm]: 17.85 (Ala-CH_3), 27.77 (CH_3), 36.58 (Phe-CH_2), 48.01 (Ala-CH), 53.42 (Phe-CH), 77.34 ($\underline{C}(CH_3)_2$), 112.76 (GCP-CH_{ar}), 126.36 (Phe-CH_{ar}), 128.08 (Phe-CH_{ar}), 129.15 (Phe-CH_{ar}), 137.41 (Phe-Cq), 155.54 (Gua-Cq), 158.43 (CO), 159.11 (CO), 172.16 (CO), 172.70 (CO). LRMS (ESI): m/z calculated for $C24H31N6O7^{+}$ $[M+H]^{+}$: 515.2; found: 515.3.

(GCP-Lys-Ala)$_3$TREN (3)

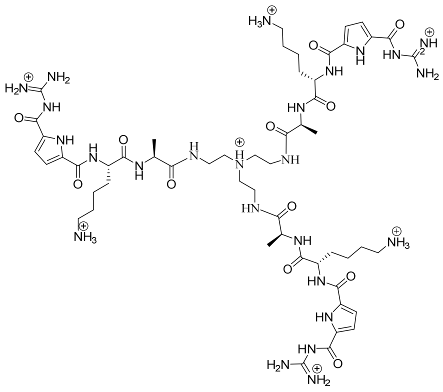

Synthesis of **3** was carried out following the procedure described on the literature.[11] It was obtained a white solid (8 mg, 27%, HPLC purity 94%). ^{1}H-NMR (500 MHz, DMSO-*d^6*) δ [ppm]: 1.22 (d, *J = 7.0 Hz*, 9H, 3 x Ala-CH_3), 1.32-1.44 (m, 6H, 3 x Lys-CH_2), 1.51-1.59 (m, 6H, 3 x Lys-CH_2), 1.60-1.66 (m, 3H, Lys-CH_2), 1.73-1.78 (m, 3H, Lys-CH_2), 2.77 (m, 6H, 3 x Lys-CH_2), 3.26 (m, 12H, 6 x CH_2), 4.21-4.26 (m, 3H, 3 x Ala-CH), 4.42-4.46 (m, 3H, 3 x Lys-CH), 6.91 (m, 3H, 3 x GCP-CH_{ar}), 7.18 (m, 3H, 3 x GCP-CH_{ar}), 7.76 (s, 9H, 3 x Lys-NH_3^+), 8.23 (s, 3H, 3 x Ala-NH), 8.51 (m, 3H, 3 x NH), 8.56 (d, *J = 7.6 Hz*, 3H, 3 x Lys-NH), 8.69 (s, 5H, 5 x NH), 11.53 (s, 3H, Gua-NH), 12.50 (s, 3H, GCP-NH). ^{13}C-NMR (125 MHz, DMSO-*d6*) δ [ppm]: 18.04 (Ala-CH_3), 22.57 (Lys-CH_2), 26.63 (Lys-CH_2), 31.22 (Lys-CH_2), 38.75 (Lys-CH_2), 48.30 (Ala-CH), 52.64 (Lys-CH), 113.61 (GCP-CH_{ar}), 115.04 (GCP-CH_{ar}), 116.95 (q, *J = 298.2 Hz*, CF_3), 125.67 (GCP-Cq), 132.22 (GCP-Cq), 155.23 (Gua-Cq), 158.78 (q, *J = 32.1 Hz*, <u>C</u>OCF_3), 159.09 (CO), 159.91 CO), 171.30 (CO). HRMS (ESI): m/z calculated for C54H89N25O12^{2+} [M+2H]$^{2+}$: 639.8556; found: 653.8453.

(GCP-Ala-Phe)$_3$TREN (4)

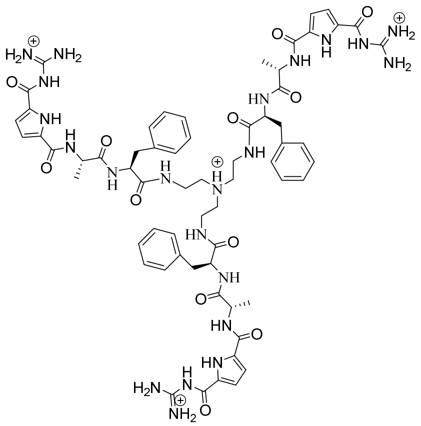

Synthesis of **4** was carried out following the procedure described on the literature.[11] It was obtained a white solid (10 mg, 25%, HPLC purity 100%).^{1}H-NMR (500 MHz, DMSO-*d^6*) δ [ppm]: 1.22 (d, *J = 7.0 Hz*, 9H, 3 x Ala-CH_3), 2.82-2.87 (m, 3H, 3 x Phe-CH_2), 3.01-3.05 (m, 3H, 3 x Phe-CH_2), 4.41-4.48 (m, 6H, 3 x Phe-CH, 3 x Ala-CH), 6.92 (m, 3H, 3 x GCP-CH_{ar}), 7.11-7.23 (m, 18H, 3 x GCP-CH_{ar}, 15 x Phe-CH_{ar}), 8.16 (m, 3H, 3 x Phe-NH), 8.47 (m, 10H, 10 x NH), 8.58 (d, *J = 6.9 Hz*, 3H, 3 x Ala-NH), 11.41 (s, 3H, Gua-NH), 12.49 (s, 3H, GCP-NH). ^{13}C-NMR (125 MHz, DMSO-*d6*) δ [ppm]: 17.58 (Ala-CH_3), 40.48 (Phe-CH_2), 49.49 (Ala-CH), 53.42 (Phe-CH), 113.63 (GCP-CH_{ar}), 115.11 (GCP-CH_{ar}), 116,99 (q, CF_3), 125.74 (GCP-Cq), 126.27 (Phe-CH_{ar}), 128,01 (Phe-CH_{ar}), 129.14 (Phe-CH_{ar}), 132.22 (GCP-Cq), 137.60, 155.16 (Gua-Cq), 158.65 (q, <u>C</u>OCF_3), 158.91 (CO), 159.82 (CO), 172.17 (CO). HRMS (ESI): m/z calculated for C63H80N22O12^{2+} [M+2H]$^{2+}$: 668.3158; found: 668.3145.

c. ^{1}H- and ^{13}C-NMR

GCP(Boc)-Phe-Ala-OH (3a)

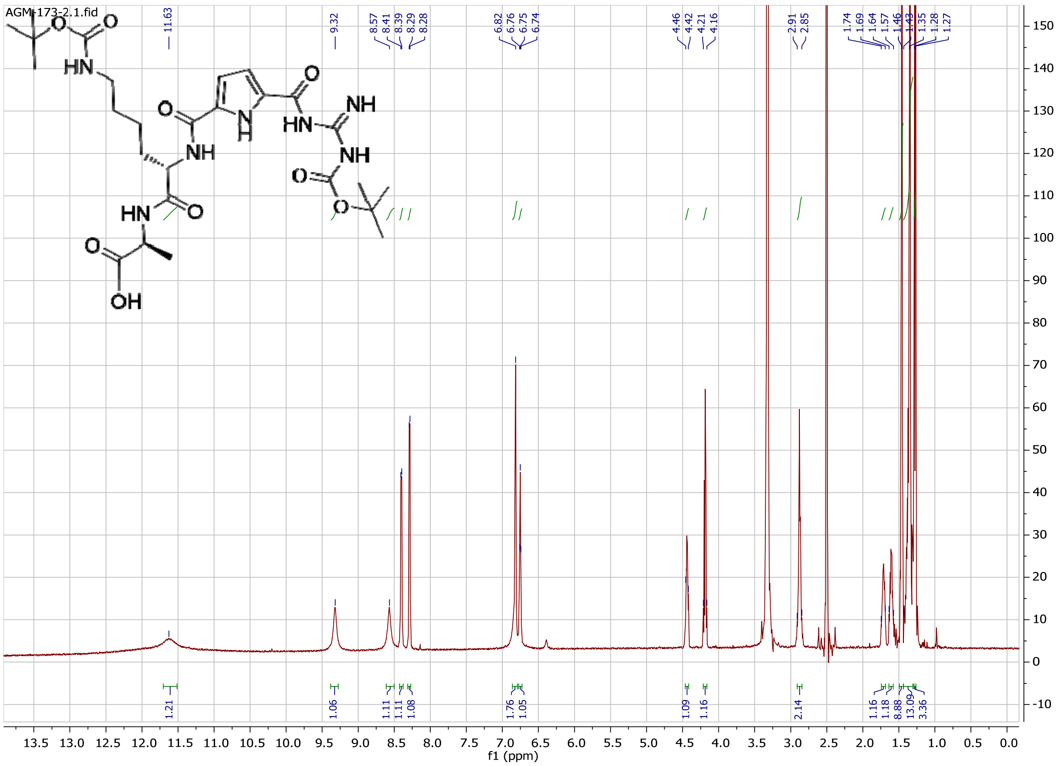

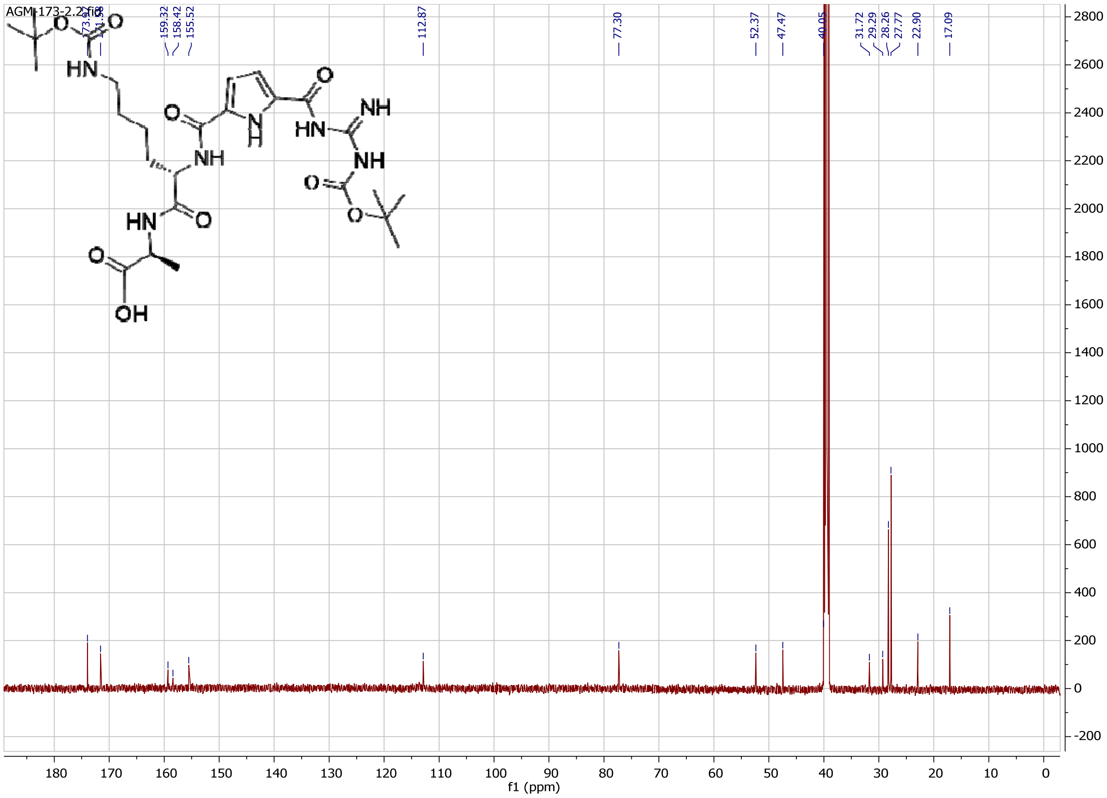

GCP(Boc)-Ala-Lys-OH (4a)

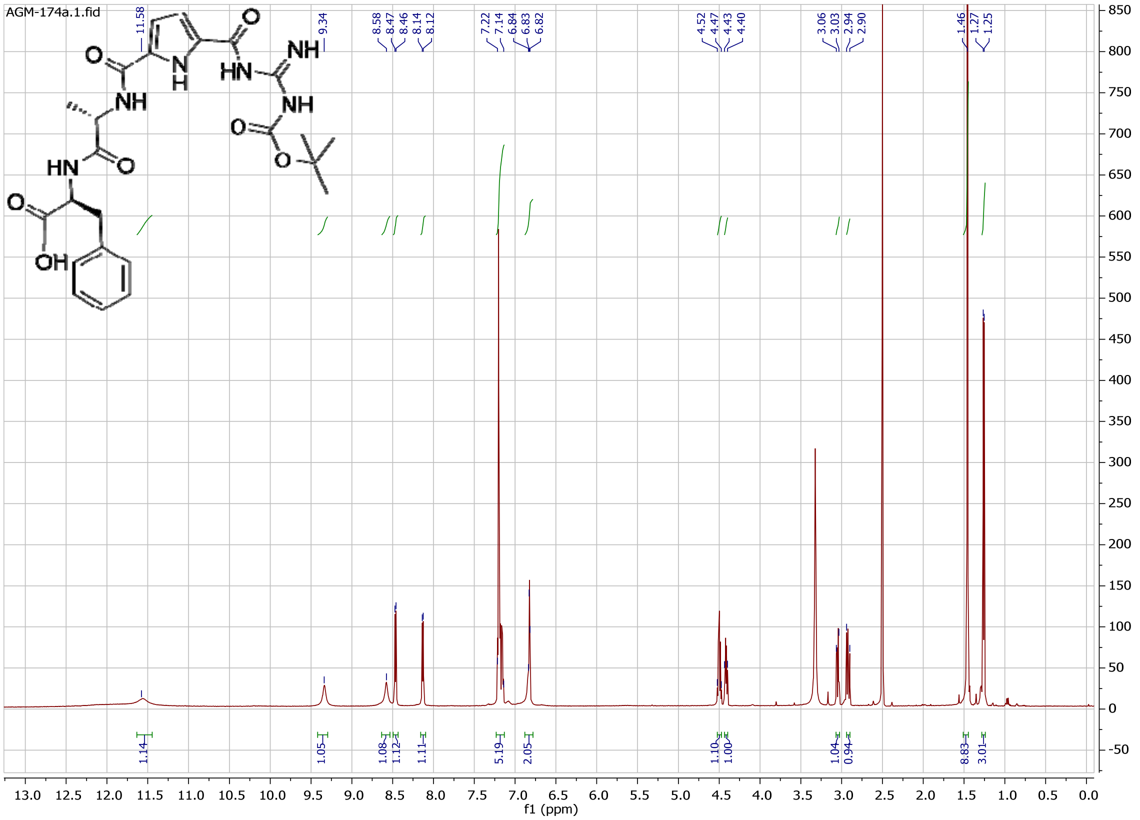

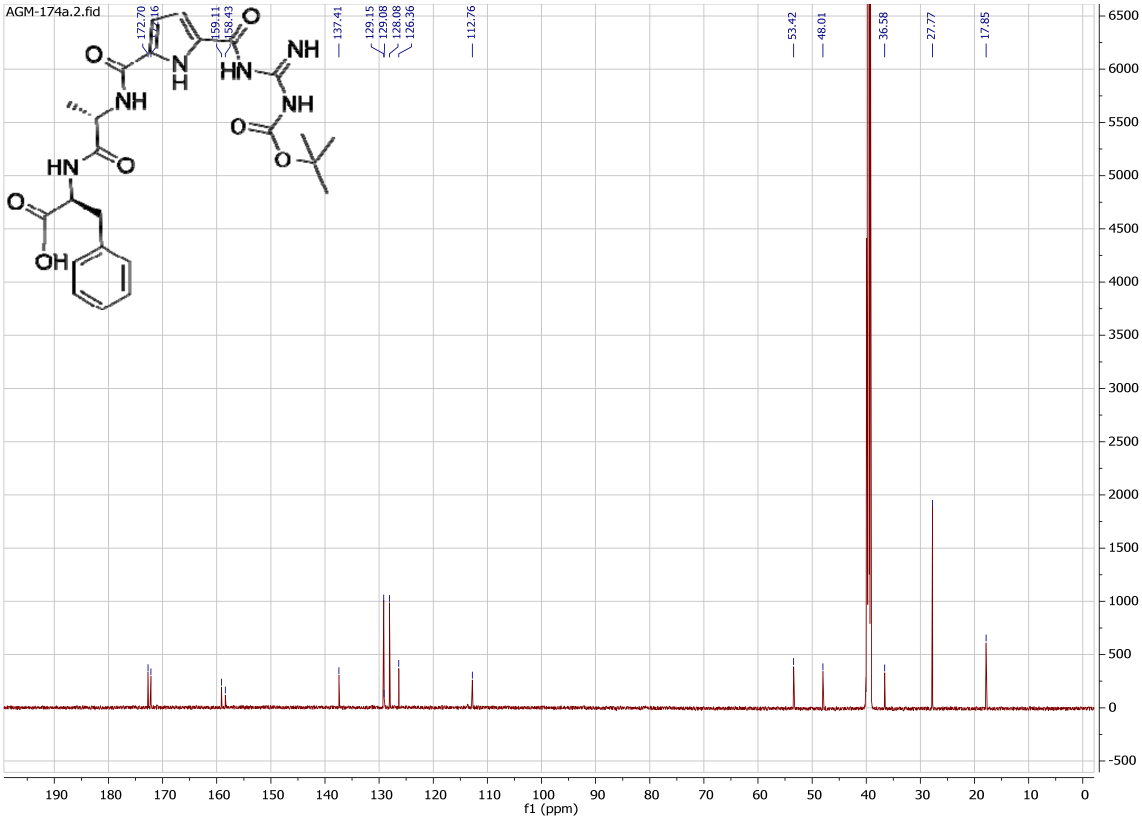

(GCP-Lys-Ala)$_3$TREN (3)

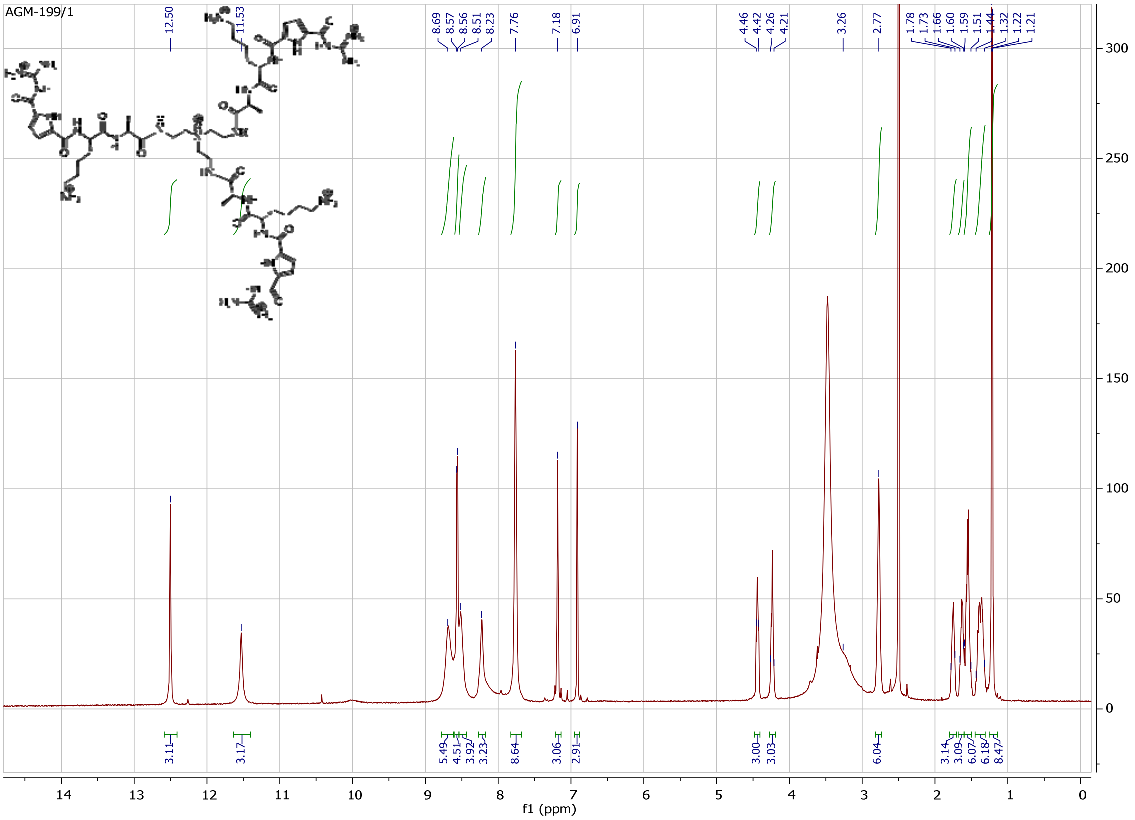

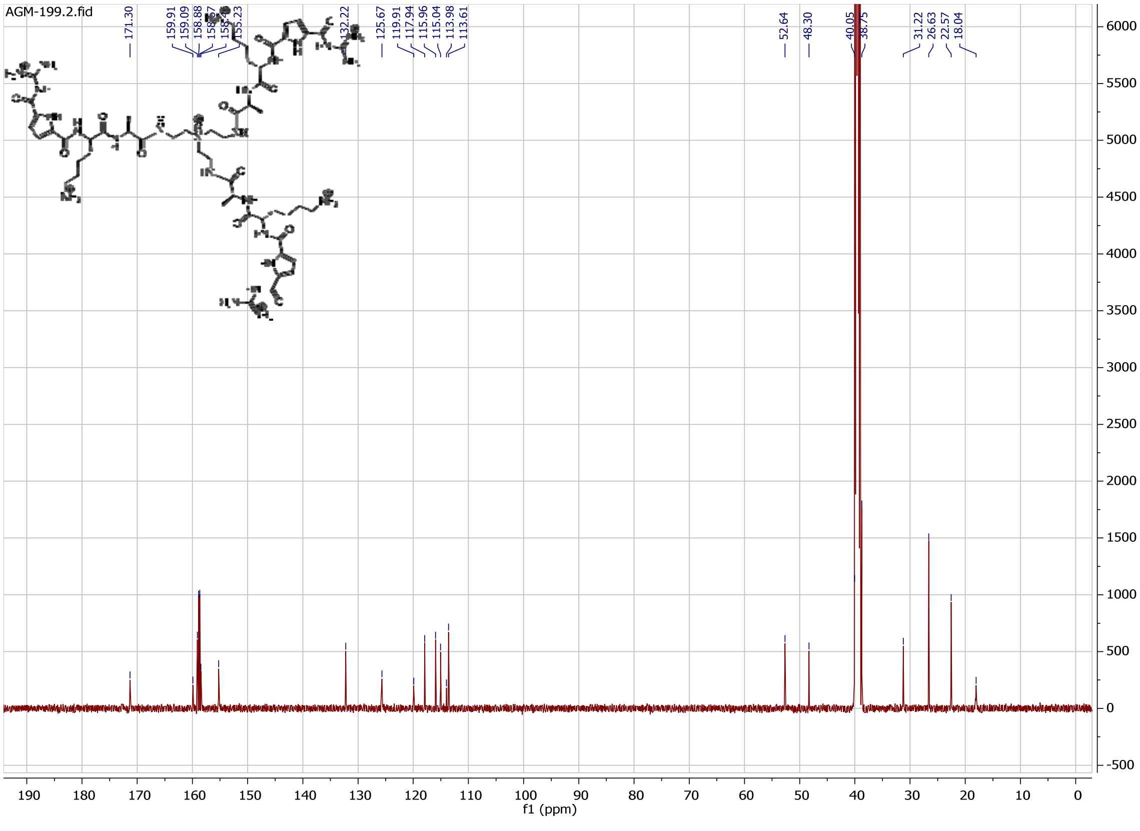

(GCP-Ala-Phe)$_3$TREN (4)

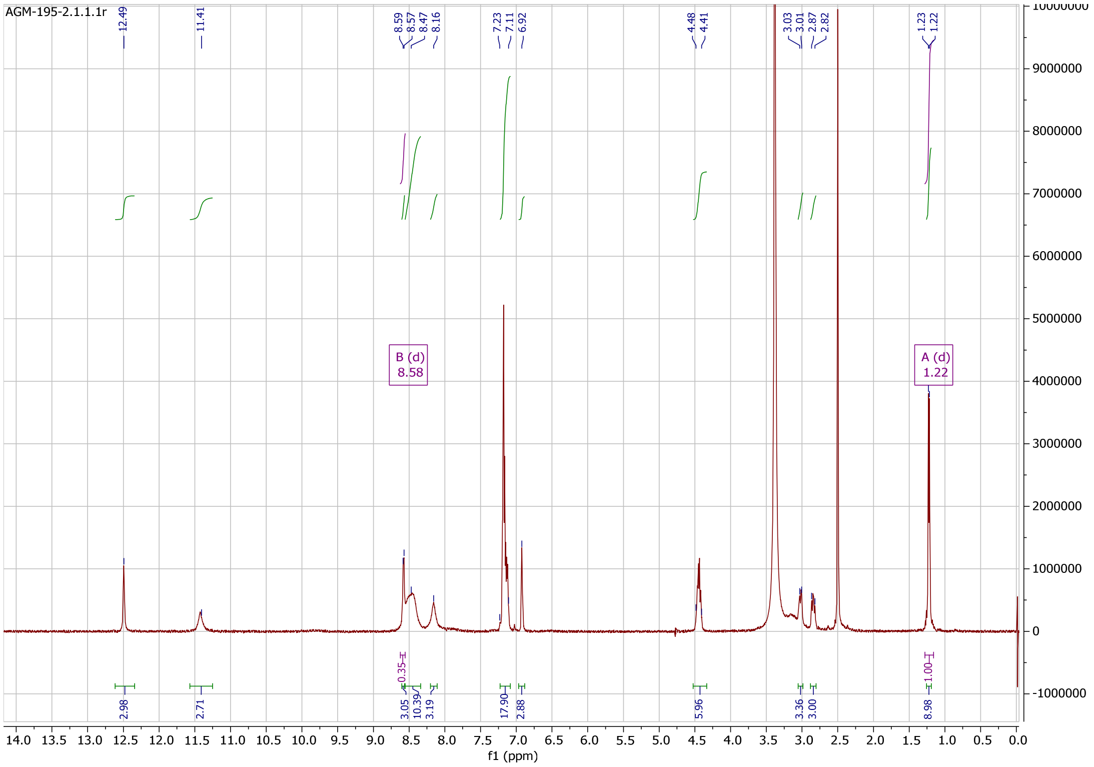

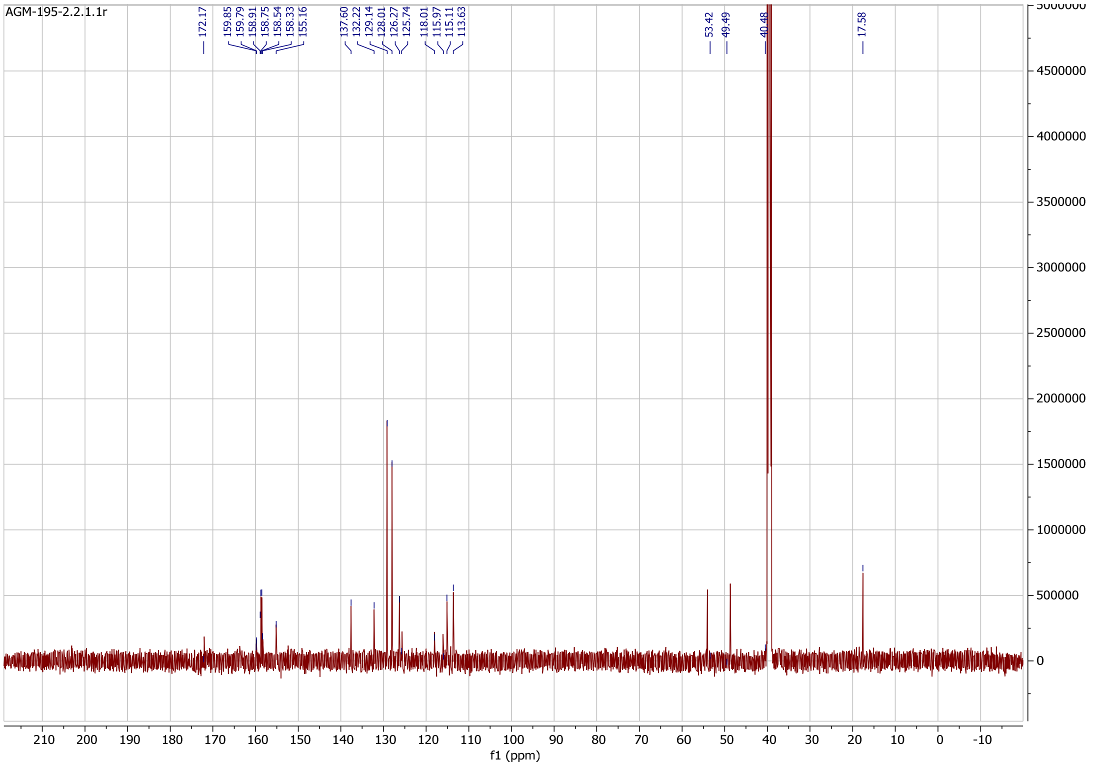

a. Purity by HPLC

Compound 3:

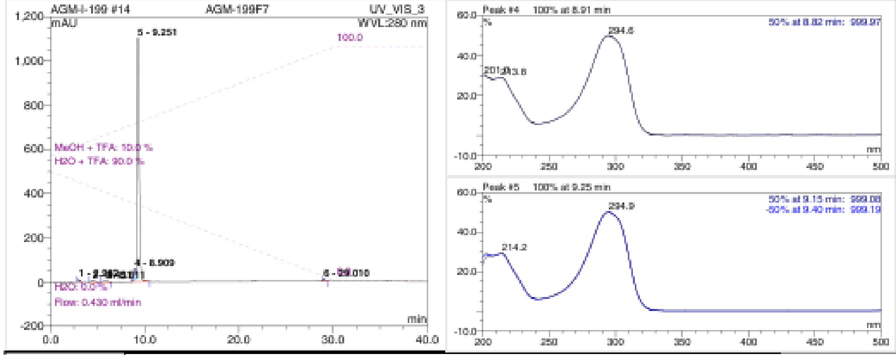

No.	Ret.Time min	Peak Name	Height mAU	Area mAU*min	Rel.Area %	Rel.Height %
1	2.98	n.a.	11.152	1.793	0.56	0.93
2	4.43	n.a.	3.636	1.557	0.49	0.30
3	5.81	n.a.	3.737	1.943	0.61	0.31
4	8.91	n.a.	60.101	12.496	3.91	5.03
5	9.25	n.a.	1103.259	299.938	93.79	92.40
6	29.01	n.a.	12.136	2.072	0.65	1.02
Total:			*1194.0205*	*319.80*	*100.00*	*100.00*

Compound 4:

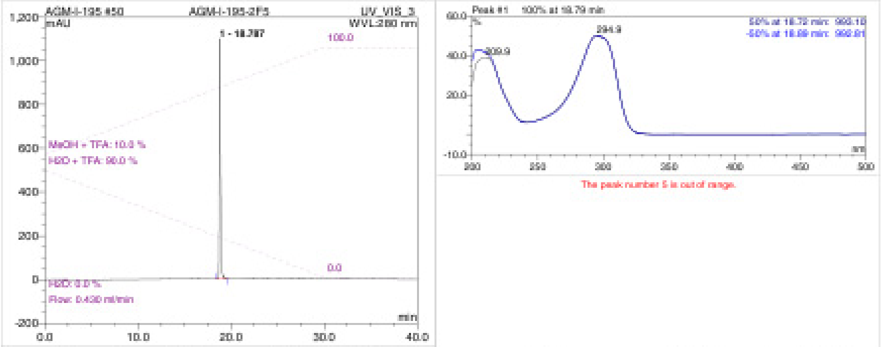

No.	Ret.Time min	Peak Name	Height mAU	Area mAU*min	Rel.Area %	Rel.Height %
1	18.79	n.a.	1096.259	205.181	100.00	100.00
Total:			*1096.2592*	*205.18*	*100.00*	*100.00*

6. References

[1] P. Thiel, L. Röglin, N. Meissner, S. Hennig, O. Kohlbacher, C. Ottmann, *Chem. Commun.* **2013**, *49*, 8468–8470.

[2] L.-G. Milroy, M. Bartel, M. A. Henen, S. Leysen, J. M. C. Adriaans, L. Brunsveld, I. Landrieu, C. Ottmann, *Angewandte Chemie International Edition* **2015**, *54*, 15720–15724.

[3] G. M. Morris, R. Huey, W. Lindstrom, M. F. Sanner, R. K. Belew, D. S. Goodsell, A. J. Olson, *Journal of Computational Chemistry* **2009**, *30*, 2785–2791.

[4] I. J. De Vries-van Leeuwen, D. da Costa Pereira, K. D. Flach, S. R. Piersma, C. Haase, D. Bier, Z. Yalcin, R. Michalides, K. A. Feenstra, C. R. Jimenez, et al., *Proceedings of the National Academy of Sciences* **2013**, *110*, 8894–8899.

[5] "Force Field Tools," can be found under http://upjv.q4md-forcefieldtools.org/REDS-Development/, **n.d.**

[6] **N.d.**

[7] W. L. Jorgensen, J. Chandrasekhar, J. D. Madura, R. W. Impey, M. L. Klein, *The Journal of Chemical Physics* **1983**, *79*, 926–935.

[8] T. Darden, D. York, L. Pedersen, *The Journal of Chemical Physics* **1993**, *98*, 10089–10092.

[9] S. Miyamoto, P. A. Kollman, *Journal of Computational Chemistry* **1992**, *13*, 952–962.

[10] J. Klett, A. Núñez-Salgado, H. G. Dos Santos, Á. Cortés-Cabrera, A. Perona, R. Gil-Redondo, D. Abia, F. Gago, A. Morreale, *Journal of Chemical Theory and Computation* **2012**, *8*, 3395–3408.

[11] S. Junghänel, S. Karczewski, S. Bäcker, S. K. Knauer, C. Schmuck, *Chembiochem : a European journal of chemical biology* **2017**, *18*, 2268–2279.
